# The Role of Eucalyptus Species on the Structural and Thermal Performance of Cellulose Nanocrystals (CNCs) Isolated by Acid Hydrolysis

**DOI:** 10.3390/polym14030423

**Published:** 2022-01-21

**Authors:** Oscar Gil-Castell, Pablo Reyes-Contreras, Pabla Andrea Barra, Regis Teixeira Mendonça, Isabel Carrillo-Varela, José David Badia, Angels Serra, Amparo Ribes-Greus

**Affiliations:** 1 Instituto de Tecnología de Materiales (ITM), Universitat Politècnica de València (UPV), Camí de Vera s/n, 46022 Valencia, Spain; ogilcastell@doctor.upv.es; 2 Department of Analytical and Organic Chemistry, Universitat Rovira i Virgili, C/Marcel·lí Domingo s/n, 43007 Tarragona, Spain; angels.serra@urv.cat; 3 Centro Nacional de Excelencia para la Industria de la Madera (CENAMAD), Pontificia Universidad Católica de Chile, Av. Vicuña Mackenna 4860, Macul, Santiago 8320000, Chile; preyes@leitat.cl; 4 Centro de Excelencia en Nanotecnología (CEN), Román Díaz 532, Providencia, Santiago 7500000, Chile; pbarra@leitat.cl; 5 Facultad de Ciencias Forestales, Universidad de Concepción, Concepción 4030000, Chile; rteixeira@udec.cl; 6 Centro de Biotecnología, Universidad de Concepción, Concepción 4030000, Chile; 7 Centro de Investigación de Polímeros Avanzados, CIPA, Avenida Collao 1202, Edificio de Laboratorios, Concepción 4030000, Chile; i.carrillo@cipachile.cl; 8 Department of Chemical Engineering, School of Engineering, Universitat de València, Avinguda de la Universitat s/n, 46100 Burjassot, Spain; jose.badia@uv.es

**Keywords:** cellulose nanocrystals (CNC), eucalyptus, crystallinity, thermal stability, kinetic analysis

## Abstract

Cellulose nanocrystals (CNCs) are attractive materials due to their renewable nature, high surface-to-volume ratio, crystallinity, biodegradability, anisotropic performance, or available hydroxyl groups. However, their source and obtaining pathway determine their subsequent performance. This work evaluates cellulose nanocrystals (CNCs) obtained from four different eucalyptus species by acid hydrolysis, i.e., *E. benthamii*, *E. globulus*, *E. smithii,* and the hybrid *En* × *Eg*. During preparation, CNCs incorporated sulphate groups to their structures, which highlighted dissimilar reactivities, as given by the calculated sulphate index (0.21, 0.97, 0.73 and 0.85, respectively). Although the impact of the incorporation of sulphate groups on the crystalline structure was committed, changes in the hydrophilicity and water retention ability or thermal stability were observed. These effects were also corroborated by the apparent activation energy during thermal decomposition obtained through kinetic analysis. Low-sulphated CNCs (*E. benthamii*) involved hints of a more crystalline structure along with less water retention ability, higher thermal stability, and greater average apparent activation energy (233 kJ·mol^−1^) during decomposition. Conversely, the high-sulphated species (*E. globulus*) involved higher reactivity during preparation that endorsed a little greater water retention ability and lower thermal stability, with subsequently less average apparent activation energy (185 kJ·mol^−1^). The *E. smithii* (212 kJ·mol^−1^) and *En* × *Eg* (196 kJ·mol^−1^) showed an intermediate behavior according to their sulphate index.

## 1. Introduction

Eucalyptus’ wood is extensively used in the global papermaking industry in different grades, such as printing, tissue or packaging [1,2]. Moreover, it is also used for chemical conversion into cellulose derivatives such as viscose, cellulose-acetate, cellulose-xanthate, carboxymethyl cellulose, and nanocellulose [3,4,5]. Diverse eucalyptus species have been reported to show significant differences in morphological, anatomical, and chemical characteristics, which define their subsequent applicability. Depending on their adaptability to the environment in which they are grown, it is possible to select a certain species based on factors such as weather resistance or growth speed. While *E. globulus* is widely applied for manufacturing Kraft pulps due to its high wood density and fast-growing plantation, other species such as *E. nitens* × *E. globulus* hybrids are used for papermaking in cold countries due to their frost tolerance. As well as this, *E. nitens* has gained interest in recent years for bleached hardwood Kraft pulp obtaining, boosted by its excellent growing rates [6]. In this regard, variations in the structure and cellulosic components of milled wood, holocellulose and α-cellulose isolated from different eucalyptus species have been described in the literature [7].

The wood of eucalyptus is mainly composed of polysaccharides (75%), followed by lignin (18–22%), other water-soluble compounds (1–2%), and extractives (<1%) [8]. Polysaccharides involve both hemicellulose and cellulose. Among them, cellulose, the most abundant renewable polymer on Earth, is composed of two β-D-glucopyranose rings rotated concerning each other to form a unit of cellobiose. Each of its monomers has three hydroxyl groups that interact with other cellulose molecules, forming intra- and inter-molecular hydrogen bonds. According to the arrangement of these bonds, the cellulose forms a rigid network, which creates the compact structure responsible for its high viscosity in solution and its high tendency to crystallize. At this supramolecular level, the cellulose structure is not uniform, and crystalline and amorphous regions can be found. The proportion of these regions depends on the raw material and the treatments to which the cellulose has been subjected to. Likewise, the molecular orientation and the H-bond network within the crystalline regions can vary widely, giving rise to different polymorphs (I, II, III, and IV), with cellulose I and II being the most studied forms [9,10,11].

The development and utilization of cellulose are of great significance for advanced biomaterials manufacture. Among the cellulose-based products, cellulose nanocrystals (CNCs) are emerging as an attractive material for multiple applications due to their renewable nature, high surface-to-volume ratio, high crystallinity, biodegradability, anisotropic performance, available hydroxyl groups for functionalization, colloidal stability, excellent mechanical properties, and good biocompatibility [12]. The CNCs are crystalline domains extracted from wood through different methods such as acid hydrolysis, enzymatic hydrolysis, or natural chemicals called deep eutectic solvents, among other approaches [13,14]. During extraction by hydrolysis, the amorphous regions may be hydrolyzed, while the crystalline regions must remain unaffected. CNCs are usually rigid, rod-like particles with a width of several nanometers (2–20 nm) and lengths up to hundreds of nanometers (100–1000 nm) [15]. Their characteristics depend on the source of the fibers and the hydrolysis conditions such as the acid concentration, temperature, and reaction time. From a technological perspective, the CNCs have been used alone or in combination with other materials. Such is the case of polymer biocomposites, in which CNCs act as fillers for mechanical reinforcement purposes, along with permeability modulator of matrices, while maintaining biodegradability [16,17,18]. Compared to other inorganic reinforcing fillers, the CNCs are widely available, easily processable due to their non-abrasive nature, and allow for high filling levels and significant cost-savings [19]. However, there are critical aspects of CNCs that need to be understood before its utilization, which determine their performance and assist to provide ubiquitous properties. For example, morphology, aspect ratio, and functionality affect the rheological properties of CNCs. Moreover, crystallinity is a critical feature due to its direct connection to strength/toughness modulus, which is a desirable characteristic for composite applications. Although research has been carried out regarding the modification of cellulose for advanced biomaterials manufacture [20,21,22,23], there are few studies in the literature dealing with the structural variation of the CNCs isolated from different species and their influence on the course of chemical reactions for derivatives production.

Carrillo et al. studied cellulosic components isolated from seven eucalyptus varieties grown under the same conditions [7]. The results showed significant correlations for the crystalline index (*CrI*), as obtained by X-ray diffraction (XRD), versus the peak of the derivative thermogravimetric curve (DTG) in wood samples and the DTG peak versus the crystallite size in alpha-cellulose samples. These structural and thermal differences can determine the production of cellulose derivatives from such species. During the production of derivatives, cellulose is modified by substitutions at the hydroxyl groups present in the C2, C3, and C6 positions of anhydroglucose units. This capacity to substitutions of hydroxyl groups is known as cellulose reactivity. Indeed, in another study dealing with cellulose from bleached kraft pulps of different eucalyptus species upgraded to dissolving pulp, they demonstrated that *E. benthamii* and *E. nitens* were the less reactive pulps, whereas other species such as *E. globulus* or the hybrid *E. nitens* × *E. globulus* (*En* × *Eg*) were more reactive [24]. The accessibility is an intrinsic property of the pulp, which depends on several chemical and structural features that could be roughly defined as the ease with which the reaction sites (functional groups) can be reached by the chemical agents [25]. The accessibility of cellulose fibers is critical for the reactivity of cellulosic pulps, and it is affected by many other inter-related and complex factors as fiber morphology, cell wall architecture, pore system, surface area, supramolecular structure, chemical composition, and molecular weight [26,27]. In this area, and considering the growing demand for cellulose derivative materials such as CNCs for sustainable industrial applications, more studies dealing with its quality and subsequent behavior are required.

This work aims to understand the structural and thermal performance of cellulose nanocrystals (CNC) obtained from different eucalyptus species, all of them grown under the same field and plantations conditions, i.e., *E. benthamii*, *E. globulus*, *E. smithii*, and the hybrid *En* × *Eg*. The physicochemical properties of the CNCs were evaluated to determine similarities and differences among the different species. In particular, the assessment of composition using spectroscopy, the evaluation of surface morphology through microscopy, the study of the crystalline structure employing diffraction and spectroscopic techniques, and thermal properties using both differential scanning calorimetry and thermogravimetry was carried out. In this regard, the assessment of the chemical composition, the crystalline structure, and the detailed evaluation of the thermal stability of CNCs may offer knowledge on their compatibility, reinforcing ability and stability during application, and also on the behavior in the course of preparation of thermoplastic-based biocomposites at high temperatures [28]. Specifically, the crystalline arrangement and the chemical composition are in turn the most influencing parameters during thermal decomposition and may be subsequently affected by the cellulose source and also by the extracting method [29]. Although the pyrolytic decomposition of CNCs extracted from mango seeds, Kraft pulp, cellophane, corn stalk or curaua fibers has been reported in the literature [30,31,32,33], the comparative evaluation of the apparent activation energy (*Ea*) of CNCs from different eucalyptus species during thermal decomposition has not been yet reported. Therefore, Kissinger, Flynn–Wall–Ozawa (FWO), and Kissinger–Akahira–Sunose (KAS) methods were used to assess the apparent activation energy (*Ea*) of such CNCs during thermal decomposition.

## 2. Materials and Methods

### 2.1. Materials

#### 2.1.1. Eucalyptus Wood Samples

The eucalyptus species corresponding to *E. benthamii*, *E. globulus*, *E. smithii*, and a hybrid of *E. nitens* × *E. globulus* (labelled as *En* × *Eg*), grew under the same field and plantations conditions and were provided by Chilean forestry located at the Biobío region (Southern Chile).

#### 2.1.2. Chemicals and Reagents

Sodium hydroxide (NaOH, ≥98%, pellets (anhydrous)), sodium sulphide nonahydrate (Na_2_S ≥ 98%,), sulfuric acid (H_2_SO_4_, 95–98%), and glacial acetic acid (CH_3_CO_2_H, 100%), sodium chlorite (NaClO_2_, 80%) were purchased from Sigma Aldrich (St. Louis, MO, USA) and Merck (Darmstadt, Germany). Deionized water was obtained from a Millipore Milli-Q system (Milford, MA, USA) to prepare all the aqueous solutions.

#### 2.1.3. Kraft Pulping and Bleaching

Kraft pulping was performed in a rotatory digester equipped with four independent Regmed reactors (Osasco, Brazil) with a volume of 1.5 L. For each reaction, wood chips (100 g on a dry basis) with average size 30 × 20 × 0.4 mm^3^ and cooking liquor (400 mL) with 18% active alkali (AA) and 30% sulphidity (both expressed on NaOH basis) were used. Heating time to the maximum temperature (165 °C) was 90 min and the H-factor was 800. The resulting material from each cooking was disintegrated, and pulps were screened on a 0.2 mm slot screen. The pulp was then centrifuged to 35% consistency and weighted. The exact moisture was determined, and the screened pulp yield was calculated.

Pulp bleaching was performed by chlorite delignification in an acidic media using a NaClO_2_ charge of 10% oven dry-pulp (ODP) basics at 5% pulp consistency. Acetic acid was added to acidify the mixture. The reaction was carried out in sealed poly(ethylene) bags immersed in a thermostatic water bath at 90 °C for 2 h. Finally, bleached pulps were obtained after filtration, washed with abundant distilled water, and centrifuged to 35% consistency.

### 2.2. Characterization of Pulp

Eucalyptus pulps (300 mg) were hydrolyzed with 72% H_2_SO_4_ (3 mL) at 30 °C for 1 h. Then, the acid was diluted to 4% and the mixture was heated at 121 °C, 1.1 atm, for 1 h. The resulting material was cooled and filtered through a porous glass filter. Solids were then dried to a constant weight at 105 °C and determined as insoluble lignin. The soluble lignin concentration in the filtrate was determined through UV-spectroscopy by measuring the absorbance at 205 nm, using the value of 110 L·g^−1^·cm^−1^ as the absorptivity. The concentration of monomeric sugars and acetic acid in the soluble fraction was determined via high-performance liquid chromatography (HPLC) in a setup from LaChrom-Merck-Hitachi (Tokyo, Japan) equipped with a refractive index detector and Bio-Rad Aminex HTX-87H column (Hercules, CA, USA) at 45 °C, a mobile phase of 5·10^−3^ mol L^−1^ of H_2_SO_4_ and a flow rate of 0.6 mL min^−1^. Glucose and xylose were used as external calibration standards. The glucans content was calculated by multiplying the glucose content by 0.90; the xylans content was obtained from the xylose content by multiplying by 0.88; and the acetyl groups content was calculated by multiplying the acetic acid content by 0.70 [34].

### 2.3. Production of Cellulose Nanocrystals (CNCs)

CNCs from eucalyptus Kraft pulps were prepared by sulfuric acid hydrolysis using the protocols published by Chen et al. [35], and Bondeson et al. [36], with some modifications. Briefly, a H_2_SO_4_ solution (55% wt.) was used with a reaction temperature of 60 °C for 45 min under constant magnetic agitation. All the acid hydrolysis experiments were conducted using a 1:40 fiber to acid solution ratio. The reaction was stopped with cold distilled water and the reaction products were washed three consecutive times by centrifugation (10 min, 12,000 rpm). The resulting suspensions were extensively dialyzed for 5 days with a membrane of 12 kDa against distilled water to a constant pH. Finally, the samples were sonicated in an iced-water bath to avoid heating of the suspension at 750 W for 10 min using pulses (10/15) and centrifuged for 10 min at 12,000 rpm. Thus, the resulting supernatant containing the isolated CNCs was kept at 4 °C. Afterwards, highly concentrated aqueous solutions of such CNCs (1 g·L^−1^) were solvent-casted in circular molds and the water allowed to evaporate until constant mass and consistent films with a diameter of 5 cm and a thickness of 15 µm were obtained. They were stored in a desiccator at room temperature and were the basis for further characterizations.

### 2.4. Characterization of Cellulose Nanocrystals

#### 2.4.1. Chemical Oxygen Demand (COD)

Chemical oxygen demand (COD) measurements were used to determine CNCs yields according to the procedure described by Wang et al. [37]. The colorimetric COD expresses the amount of oxygen originating from potassium dichromate that reacts with the sample. The samples were oxidized with a hot sulfuric solution of potassium dichromate. Microcrystalline cellulose was used for calibration and further calculation. A proper aliquot was taken and added in the COD reaction tubes from Merk Spectroquant COD cell test (Darmstadt, Germany). The tubes were thoroughly mixed and incubated for 2 h at 148 °C using a Merck Spectroquant TR320 thermoreactor (Darmstadt, Germany). Once the samples were cooled down to room temperature, the absorbance at 600 nm was measured in a Merck Spectroquant Pharo 300 spectrophotometer (Darmstadt, Germany), and the COD was determined by interpolation using calibration curves with Avicell PH-101 cellulose from Sigma-Aldrich (St. Louis, MO, USA).

#### 2.4.2. Field-Emission Scanning Electron Microscopy (FE-SEM)

The surface topology was analyzed by field-emission scanning electron microscopy (FE-SEM) in a Zeiss Ultra 55 equipment (Oberkochen, Germany). They were prepared in two ways: either small pieces of the films (1 cm diameter) were placed on the sample holders with double-sided adhesive carbon tape; or aqueous low-concentrated dispersions (2 mg·L^−1^) of the different samples were prepared, sonicated, and directly deposited onto clean circular coverslips, and water evaporation was allowed overnight. Then, all the samples were sputter-coated with a platinum layer for 15 s using a Leica EM MED020 sputter coater (Wetzlar, Germany). Finally, the micrographs were taken using a working distance between 3 and 4 mm, a voltage of 2 kV, and magnification of 100,000×. The size and distribution of the different samples were measured from the average of random locations (*n* = 100) through the software Image J^®^ 1.52p.

#### 2.4.3. Fourier-Transform Infrared Spectroscopy (FTIR)

Fourier-transform infrared spectroscopy (FTIR) was used to study the different functional groups of eucalyptus nanocrystals and to evaluate structural changes. All the experiments were conducted using an Agilent Cary 630 FTIR spectrometer (Santa Clara, CA, USA) in the attenuated total reflectance mode (ATR). The solvent-casted film samples were dried before measurement in an oven at 50 °C for 24 h. The analyses were performed in the wavelength range from 4000 to 650 cm^−1^ with a 4 cm^−1^ resolution and an average of 64 scans. From the intensities of bands at 1430 and 897 cm^−1^, the infrared crystallinity index (*ICI*) was calculated with Equation (1), as proposed by O’Connor et al. [38]. Then, Nelson and O’Connor proposed a new infrared crystallinity ratio (*ICR*), considering the bands at 1372 and 2900 cm^−1^ assigned to a C–H bending mode and C–H and CH_2_ stretching, according to Equation (2) [39].

(1) $$ICI\left(\%\right)=\frac{A_{1430}}{A_{897}}\times 100$$

(2) $$ICR\left(\%\right)=\frac{A_{1372}}{A_{2900}}\times 100$$

#### 2.4.4. X-ray Diffraction (XRD)

An X-ray diffraction instrument PANalytical Cubix with a PANalaytical X’Celerator detector (Malvern, Worcestershire, UK) was used to determine the crystal size and the crystallinity degree of the samples. Dried film specimens of 100 mm^2^ were considered for the analysis. The CuKα radiation (λ = 1.5418 Å) was generated with a tension of 45 kV and a current of 35 mA and then monochromatized by using a Ni filter of 20 μm. The experiment was measured and recorded in a reflection mode at the angular range of 2–90° (2θ) and a rate of scanning of 0.05° per 10 s. Results were analyzed using the X’Pert Highscore software. An average spectrum of three different specimens was considered as representative for each sample.

To determine the mean size of the crystalline domains, the Scherrer’s equation was exploited according to Equation (3), where *K* is a dimensionless shape factor (0.96), *λ* is the X-ray wavelength (0.154 nm), *β* is the line broadening at half the maximum intensity after subtracting the instrumental line broadening in radians and *θ* is the Bragg angle. The peak of 2*θ* = 22.6° was observed to calculate the crystal size, which represented the diffraction of the (002) plane.

(3) $$L_{hkl}=\frac{K\lambda }{\beta \cos\theta }$$

The crystallinity index (*CrI*) was calculated employing the method proposed by Segal et al. according to Equation (4), where *I*_002_ is the maximum density of the (002) reflection at 2*θ* = 22.6° and *I_am_* is the intensity diffraction of the amorphous band at 2*θ* = 18.5°.

(4) $$CrI\;\left(\%\right)=\frac{\left(I_{002}-I_{am}\right)}{I_{002}}\times 100$$

#### 2.4.5. Differential Scanning Calorimetry (DSC)

The calorimetric data were obtained using a Mettler–Toledo DSC 820e differential scanning calorimeter (Columbus, OH, USA). The solvent-casted film samples, with a mass of around 5 mg, were introduced into 40 μL aluminum crucibles perforated on top. The method of analysis consisted of a heating scan between 25 °C and 375 °C with a rate of 10 °C·min^−1^. The test was carried out under an inert atmosphere of nitrogen with a flow rate of 50 mL·min^−1^. The samples were analyzed in triplicates with the aid of the STARe^®^ software, and the averages were taken as representative.

#### 2.4.6. Thermogravimetric Analysis (TGA)

The thermal stability was assessed through a Mettler–Toledo TGA 851 thermogravimetric analyzer (Columbus, OH, USA). The solvent-casted film samples, with a mass between 3 and 5 mg, were introduced into 70 μL alumina capsules. Next, they were subjected to a dynamic assay, based on a heating segment from 25 °C to 800 °C with a heating rate (*β*) of 5, 10, 20, and 30 °C min^−1^. The analyses were carried out under an inert atmosphere with a feeding rate of argon at 50 mL per min^−1^. The decomposition onset and endset temperatures (*T_o_*, *T_e_*) were obtained by a tangential intercept method onto the thermogravimetric (TG) curves. The peak temperatures were obtained from the maximum of the derivative thermogravimetric curves (DTG), i.e., the maximum decomposition rate. The samples were analyzed in triplicates with the aid of the STARe^®^ software, and the averages were taken as representative.

#### 2.4.7. Kinetic Analysis for the Thermal Decomposition

The apparent activation energy (*Ea*) of the thermal decomposition process was calculated through kinetic analysis from the obtained thermogravimetric results at different heating rates (*β*) [40,41,42,43]. Different approaches were applied, including Kissinger [44], Flynn–Wall–Ozawa (FWO) [45,46], and Kissinger–Akahira–Sunose (KAS) [47] methods.

First, the *Ea* was estimated considering the method proposed by Kissinger, based on the temperature at maximum decomposition rate (*T_p_*), the model for which is given by Equation (5).

(5) $$\ln\frac{\beta }{T_{p}^{2}}=-\frac{Ea}{R\cdot T_{p}}+\ln\;\left(\frac{A\cdot R}{Ea}\cdot f^{\prime }\left(\alpha \right)\right)$$

where *β* is the heating rate (K·min^−1^), *R* is the gas constant (8.314 J·K^−1^·mol^−1^), *Ea* is the apparent activation energy (J·mol^−1^), *A* is the pre-exponential factor (min^−1^) and *T_p_* (K) is the peak temperature of the main decomposition stage in the DTG curve. According to this method, the activation energy can be calculated from the slope of the plots of ln (*β*/*T_p_*^2^) versus the reciprocal of temperature at the maximum reaction rate (1/*T_p_*), for the different experiments at constant heating rate. Therefore, the activation energy can be estimated regardless of the reaction mechanism and conversion.

Then, both isoconversional FWO and KAS models were applied, which involve the evaluation of the thermal decomposition as a function of the conversion degree (*α*). Neither of these methods require knowledge of the particular thermal degradation mechanism.

(6) $$\log\beta =\log\frac{A\cdot Ea}{R\cdot g\left(a\right)}-2.315-0.4567\frac{Ea}{R\cdot T_{\alpha }}$$

Finally, the Kissinger–Akahira–Sunose (KAS) method for a multi-heating rate application is based on Equation (7). Through this method, the apparent activation energy can be estimated from the slope of the plot of ln (*β*/*T*^2^) versus 1/*T* at a given conversion degree.

(7) $$\ln\frac{\beta }{T_{\alpha }^{2}}=\ln\left(\frac{A\cdot R}{Ea\cdot g\left(\alpha \right)}\right)-\frac{Ea}{R\cdot T_{\alpha }}$$

## 3. Results and Discussion

### 3.1. From Eucalyptus Pulp to Cellulose Nanocrystals (CNCs)

Eucalyptus Kraft pulps were prepared and their yield and chemical composition were determined. As gathered in Table 1, the unbleached Kraft pulps yields ranged between 49% and 57%, with the species *E. benthamii* being the one with the lowest yield. After the bleaching stage, the yields of solids generally decreased, reaching values of 45% to 53%, since not only residual lignin is removed but also some low molar mass xylans and glucans from polysaccharides fraction are detached. The obtained yield and chemical composition found in this study are in agreement with the values reported in the literature for eucalyptus wood with similar pulping conditions [48].

Cellulose nanocrystals (CNCs) were subsequently isolated, and the obtaining yields were calculated. Table 1 gathers significant differences in the yield for the different eucalyptus species. While the *E. globulus* showed the lowest CNC yield, around 15%, the yield for the hybrid *En* × *Eg* reached almost 25%, based on COD measurements. Although a wide variety of CNC yield can be found in the literature according to specific conditions and CNC source [37,49,50], these values generally agreed with other studies using a similar CNC production strategy from eucalyptus, based on sulfuric acid hydrolysis with 55% wt. H_2_SO_4_, 60 °C and 45 min, such as reported by Chen et al. with yields near to 18% [35]. The variances found in the CNC yield can be ascribed to the differences in the properties of cellulose from the diverse eucalyptus type, as reported by Carrillo et al. in wood and holocellulose isolated from the same species [7].

### 3.2. Cellulose Nanocrystals (CNCs)

#### 3.2.1. Morphology

**Figure 1:**
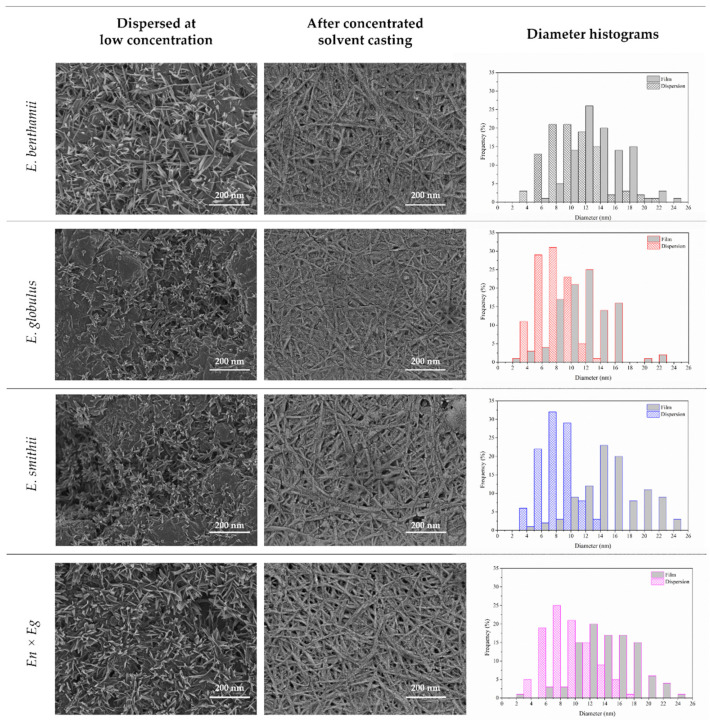
Surface electron micrographs and diameter distribution of the CNCs obtained from the different eucalyptus species.

It is well known that the origin of cellulose microfibrils and the hydrolytic conditions strongly affect the geometric dimensions and morphology of CNCs. For this reason, the surface morphologies of the CNCs obtained from the different eucalyptus species were evaluated, and the size distributions in terms of diameter were calculated. In particular, the morphology of the low-concentrated dispersions was studied together with the surface of the solvent-casted films. The obtained surface micrographs along with the diameter histograms are shown in Figure 1.

On the one hand, the deposition of low-concentrated dispersions allowed the cellulose nanocrystals to be clearly observed, with an appearance of individual separated CNCs with average diameters of 9.5, 6.6, 7.2 and 8.5 nm for the *E. benthamii*, *E. globulus*, *E. smithii* and *En* × *Eg*, respectively. For these CNCs, the aspect ratio was between 5 and 10, in line with the standardized definitions for such nanoparticles [51]. On the other hand, after the preparation of the solvent-casted films from highly concentrated solutions, a random distribution of individual and aggregated stacked shapes with respective average diameters of 14.8, 12.6, 16.7 and 15.5 nm were found in all cases [33]. The anionic stabilization via the repulsion forces of electrical double layers caused by the surface functionalization during hydrolysis may have prevented the aggregation of cellulose nanocrystals and explained the formation of the self-assembled structures [52]. Although in these pictures a fibrous appearance could be identified in the first plane, no signs of network-like structures or inter-particle entanglements (or both) typical of cellulose nanofibers and nanofibrils (CNF) could be detected [51]. Moreover, fracture signs transversal to these fibrous units could be perceived, which have been reported as signs of an intermediate semi-fibrous stage during the generation of cellulose nanocrystals by acid hydrolysis [53]. At this point, the authors hypothesize that during the hydrolytic process for the preparation of CNCs, such mentioned intermediate species remained in the dissolution, and given their lower density and higher size, they were deposited on top, while the denser CNCs with lower size were deposited in the inner part of the film overlapping the visualization of the CNCs after the preparation of the solvent-casted films. According to the dimensions and morphology the approximated aspect ratio in the range from 15 to 60 for all the species lies in the range of long rod-shaped CNCs, with great potential for being used as good reinforcing agents in the preparation of bionanocomposites with improved mechanical properties [51,54].

Overall, it can be remarked that the lower diameter for the CNCs from *E. globulus* species were both in the highly dispersed CNCs and in the solvent-casted films. The progression of the hydrolytic reaction in the *E. globulus* species may have reached a greater extent and, consequently, resulted in lower diameters of the CNCs. In comparison to results found in the literature, the dimensions of the obtained CNCs were comparable to those found by other authors [19,55]. For instance, Wang et al. found diameters between 10 and 17 nm for CNCs from eucalyptus solid residues [37], Aguayo et al. reported diameters of around 40 nm for CNCs obtained using severe hydrolysis conditions from rejected fibers originated in the Kraft pulping process [56] and Duarte Urueña et al. reported diameters varying from 4 to 15 nm in eucalyptus-based residues [57]. Then, Fortunati et al. found diameters in the range of 5 to 10 nm in CNCs isolated from commercial microcrystalline cellulose [58], Wulandari et al. achieved CNCs from sugarcane bagasse with an average diameter of around 100 nm [59], and Kusmono et al. produced CNCs from ramie fibers with diameters from 4 to 7 nm [60].

#### 3.2.2. Chemical Structure

**Figure 2:**
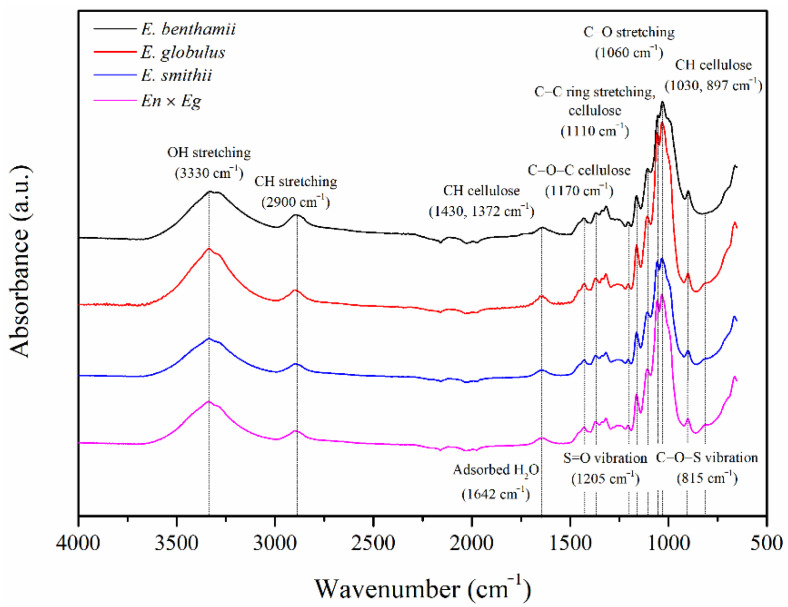
FTIR spectra of the CNCs obtained from the different eucalyptus species.

To evaluate possible functionalization caused by the acid hydrolysis process, the chemical structure of the cellulose nanocrystals (CNCs) was analyzed by Fourier-transform infrared spectroscopy (FT-IR). The acquired FTIR spectra are shown in Figure 2.

All the spectra were similar regardless of the eucalyptus species, showing the characteristic peaks of crystalline nanocellulose [56]. FTIR results corroborated that the lignin and hemicellulose were completely removed during the isolation process of the CNCs, given the absence of their characteristic peaks at 1250 cm^−1^, 1510 cm^−1^, and 1730 cm^−1^ related to the stretching vibrations of C–O of the aryl group in lignin, C=C of the aromatic ring in lignin, and C=O of carboxylic groups of hemicellulose and lignin, respectively [59,61,62].

The most dominant spectral bands in the spectra of the CNCs were located at 3330 cm^−1^ and 1040 cm^−1^ and are associated with the stretching vibration of –OH and C–O ether groups, respectively. The peak around 1640 cm^−1^ is due to the O–H bending of adsorbed water molecules, which were difficult to remove even after drying given the strong cellulose–water interaction. Peaks observed at around 2900 cm^−1^ are associated with the asymmetric and symmetric vibration of –CH_2_ groups. Scissoring motion of –CH_2_ in cellulose could be identified in the range from 1300 to 1400 cm^−1^. The peak at ~890 cm^−1^ is due to β-glycosidic linkages. Finally, the presence of sulphate groups was perceived at 1205 cm^−1^ given the S=O vibration and the small shoulder at 814 cm^−1^ caused by C–O–S group vibration.

These are remnant cues that come from the hydrolytic agent (H_2_SO_4_) used for the isolation of CNCs that may have established sulphate esters with the hydroxyl groups on their surface during hydrolysis [60]. In this regard, it is important to remark that the small peak of the C–O–S group vibration was less relevant for the *E. benthamii* species, which may be less reactive and more resistant to substitution reactions during hydrolysis. For comparative purposes, a sulphate index was established according to Equation (8), relating the absorbance of the signal at 814 cm^−1^ of sulphates with the band at 2900 cm^−1^ of the C–H and CH_2_ stretching, considered as a reference as it remains unaffected in the cellulose backbone.

(8) $$Sulphate\;index\;\left(\%\right)=\frac{A_{814}-A_{2900}}{A_{2900}}\times 100$$

The obtained values are gathered in Table 2. In particular, the *E. benthamii* was by far the less sulphated species, followed by the *E. smithii*, *En* × *Eg*, and *E. globulus* in increasing order. The degree of incorporation sulphate groups may be therefore ascribed to the dissimilar reactivity of the CNCs, as found in bleached pulps after acid hydrolysis [24] and in holocellulose and α-cellulose isolated from different eucalyptus species [7,63]. Moreover, cellulose sulphation has been reported in the literature as indicative reactions of CNC formation, so that the generation of cellulose nanocrystals can be corroborated [35,37,64].

#### 3.2.3. Crystalline Structure

**Figure 3:**
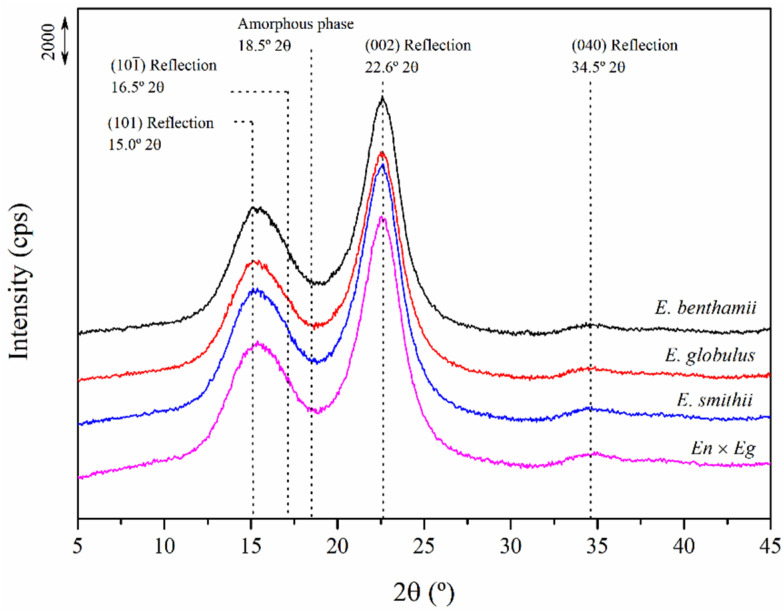
X-ray diffraction (XRD) spectra of the CNCs obtained from the different eucalyptus species.

Regarding the crystallinity evaluation, X-ray diffraction (XRD) analyses were carried out and the obtained spectra are plotted in Figure 3. Four main reflection peaks were identified at 2*θ* = 15.0°, 16.5°, 22.6° and 34.5° due to the (101), ($10\bar{1}$), (002) and (040) crystallographic planes of the monoclinic cellulose I-β lattice [65]. The highly crystalline structure of the obtained CNCs was thus corroborated and results were consistent with cellulose crystal structure I [31].

From these spectra, and considering the maximum density of the (002) reflection at 2*θ* = 22.6° characteristic of the cellulose I and the intensity diffraction of the amorphous band at 2*θ* = 18.5°, the lateral crystallite size (*L*_002_) and the crystallinity index (*CrI*) were calculated using Equations (3) and (4), respectively, and gathered in Table 3. The *CrI* of the CNC was around 65% and the *L*_002_ values varied between 10 and 11 nm regardless of the eucalyptus species. The results indicate that the crystalline structure does not present more than slight differences among the CNCs from the different species. In general, both *CrI* and *L*_002_ were higher than those of analogous wood eucalyptus counterparts due to the acid hydrolysis extraction of amorphous regions during CNC obtaining [7].

Results were comparable to those reported before for CNC produced by acid hydrolysis with sulphuric acid from eucalyptus [64,66]. Even though slightly higher values between 65 and 75% have been reported for eucalyptus CNCs with other hydrolysis approaches, the presence of such intermediate-semi-fibrous units may have prevented the obtained CNC to reach such higher crystallinity indexes [57]. Moreover, it must be considered that the hydrolysis reactions during CNC production mainly cause changes in the amorphous domains. However, it may also promote damage in the crystalline regions and, therefore, influence the overall crystallinity resulting in such moderate *CrI*. Similar values were calculated in other studies with acid hydrolysis isolation processes from other biomasses [29,33,51,60].

Despite the evaluation of the crystalline structure by XRD being the most common approach, a complimentary assessment of crystallinity through FTIR is also considered in the literature [38,39]. Therefore, the crystalline structure was studied from the FTIR spectra through some infrared crystallinity indexes defined in the literature. According to O’Connor et al., the infrared crystallinity index (*ICI*), can be obtained using Equation (1) from the ratio between the bands at 1430, assigned to crystalline areas, and 897 cm^−1^, assigned to amorphous regions [38]. Then, Nelson and O’Connor proposed a new infrared crystallinity ratio (*ICR*), shown in Equation (2), considering the bands at 1372 and 2900 cm^−1^ assigned to a C–H bending mode and C–H and CH_2_ stretching [39]. *ICI* correlates with the overall degree of order in cellulose and is defined as an empirical crystallinity index while *ICR* has been reported to be proportional to the crystallinity degree of cellulose [67,68]. Both infrared crystallinity values are gathered in Table 3.

Similar crystallinity parameters were found regardless of the eucalyptus species as indicated by the XRD, although these methods are dependent on the quantity and size of crystallites, whereas the infrared ratios are dependent upon the environment of individual molecules [39]. Only a slight increase was observed in *ICI* and *ICR* indexes for the *E. benthamii* variety, which may be correlated to the presence of sulphate groups in the CNCs and the progression of the hydrolysis reaction in the crystalline phase. The *E. benthamii* was the one with a lower sulphate index; therefore, it seemed that the higher the reactivity involving the greater incorporation of sulphates during hydrolysis, the lesser ordered structure was perceived [24].

#### 3.2.4. Thermal Properties and Thermal Stability

The thermal transitions of CNCs were assessed through differential scanning calorimetry (DSC) and the variations in the thermal stability were evaluated through thermogravimetric analyses (TGA).

**Figure 4:**
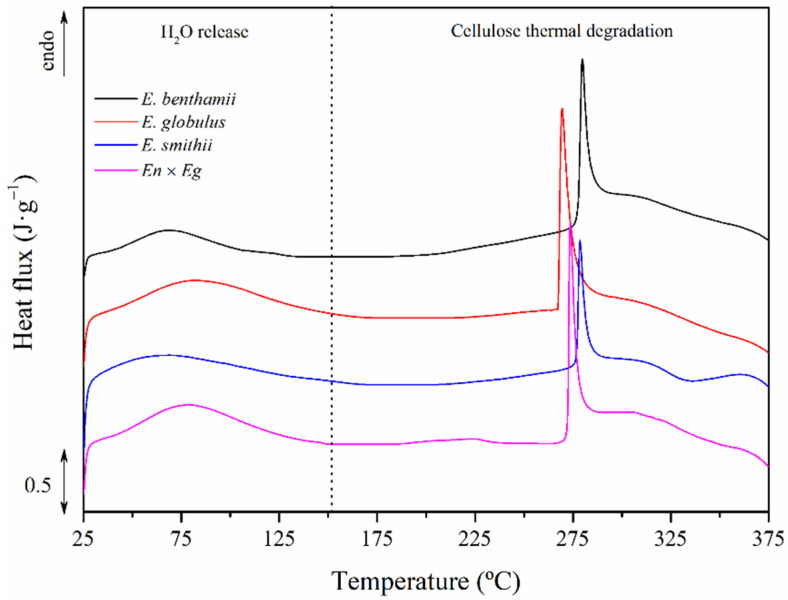
Stacked calorimetric thermograms of the CNCs obtained from the different eucalyptus species.

The calorimetric thermograms of the CNCs obtained from the different eucalyptus species are plotted in Figure 4, in which a wide endothermal transition from 25 °C to 150 °C, followed by a sharp peak in the vicinities of 275 °C with a small shoulder above 300 °C, more visible in the *E. smithii* and *En* × *Eg* species, could be observed. The peak temperatures and enthalpies for both transitions are gathered in Table 4.

The first endothermic process can be ascribed to a dehydration stage [69]. The peak for this process (*T_free_*) was located between 70 and 80 °C for the different eucalyptus species, with enthalpies (∆*h_H_*_2*O*_) ranging from 50 to 85 J·g^−^^1^. The results indicate a correlation between sulphate index and the *T_free_* and ∆*h_H_*_2*O*_ values. The CNC from *E. globulus* has the highest sulphate index and highest *T_free_* and ∆*h_H_*_2*O*_. The one from *E. benthamii* revealed the lowest *T_free_* and ∆*h_H_*_2*O*_, correlated to a lower sulphate index. Finally, those CNCs from *E. smithii* and *En* × *Eg* showed intermediate values according to their sulphate indexes. These results may suggest the contribution of sulphate groups attached to CNCs, which may have promoted structures with more capability to incorporate water.

Then, an endothermal acute transition and subsequent shoulder were found for the thermal decomposition of the crystalline nanocellulose. This peak increased while the sulphate index decreased. It was situated around 270 °C for the *E. globulus*, at 274 °C for the *En* × *Eg*, followed by the *E. smithii* at 279 °C and the *E. benthamii* at 280 °C. These emerging differences in the thermal stability of CNCs were presumable due to the effect of the sulphate groups and were examined in detail with thermogravimetric stability analyses [29,70,71].

**Figure 5:**
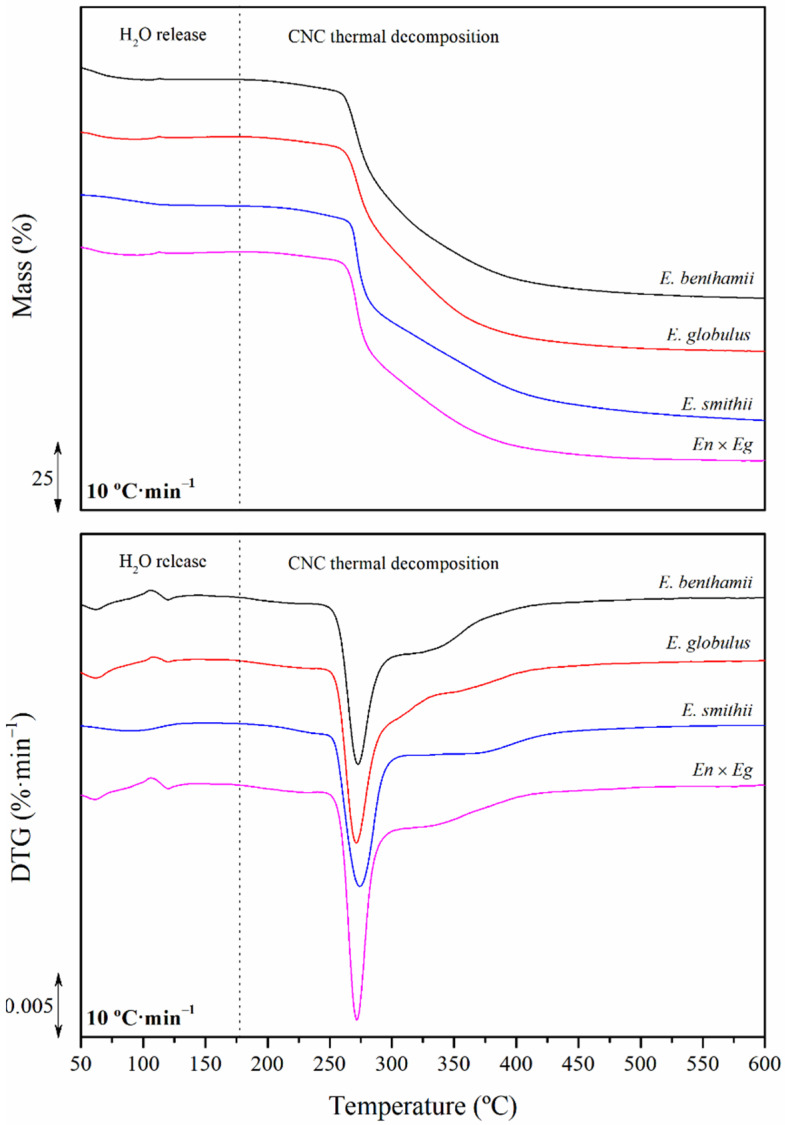
Stacked mass variation thermograms (**up**) and first derivative traces (DTG) (**down**) of the CNCs obtained from the different eucalyptus species analyzed at 10 °C·min^−1^.

The thermogravimetric thermograms with their first derivative curves obtained under inert atmosphere at 10 °C·min^−^^1^ are plotted in Figure 5. The observed stages are the two identified by the calorimetric results, and the char formation, given as the remnant residue at the end of the thermogravimetric analyses. The progressive decomposition behavior in the two stages were corroborated in the derivative thermogravimetric (DTG) curves, in which water release together with a prominent peak and a wider process at high temperatures associated with the CNC decomposition were perceived. This behavior has been previously reported for CNCs obtained from eucalyptus [57,72], but also from corn stalk [33], kenaf [63], and coconut husk [54]. The obtained thermograms were thoroughly assessed and the values of the mass loss (∆*m*) and peak temperatures (*T_H_*_2*O*_, *T_p_*) of both stages, along with the onset and endset decomposition temperatures (*T_o_*, *T_e_*) and the final residue (*R*) were calculated and gathered in Table 5.

In general, humidity below 5% was found regardless of the eucalyptus species, as suggested by the horizontal step of the first mass loss from 25 °C to 150 °C. The main release of humidity was found below 100 °C. A minor shoulder in the DTG curve could be intuited around 120 °C, due to the release of presumable bound water [73].

The next decomposition peak is from 260 to 280 °C with a shoulder from 300 to 400 °C. Although temperatures around 350 °C have been reported for the thermal decomposition of cellulose, the presence of sulphate groups in the surface of CNCs replacing the hydroxy groups may have catalyzed the decomposition and, therefore, decreased the stability of the CNCs [29]. Nevertheless, the low-temperature decomposition stage may be attributed to the degradation of amorphous regions that are more sulphated and accessible, and at higher temperatures the degradation of the non-sulphated CNCs may occur [31,74]. Similar results have been reported for CNCs isolated from corn stalk [33], kenaf [63], potato [75], and coconut husk [54]. On the other hand, these results also show that the typical decomposition stages of hemicelluloses, lignin, and other minor components below 220 °C were absent, so it was corroborated that these low thermal stability compounds were eliminated during the production of CNCs.

Although differences among the eucalyptus species were non-critical, some observations can be highlighted, especially correlated to the presence of sulphate groups. The CNCs from *E. benthamii* and *E. smithii* showed a lower water retention ability (∆*m_H_*_2*O*_) than the *En* × *Eg* and *E. globulus* ones. As well as this, higher peak temperatures (*T_H_*_2*O*_) were found for the more sulphated and less crystalline CNCs [60]. Afterwards, the thermal stability, given by *T_o_*, *T_p_*, and *T_e_*, generally followed the sequence *E. benthamii*, *E. smithii*, *En* × *Eg*, and *E. globulus*, according to the mentioned sulphate index. Furthermore, the shoulder above 300 °C was less relevant as the sulphate index increased, correlated to the prevalence of a less crystalline structure with lower crystallite size [76].

The most sulphated CNCs showed lower thermal stability due to the catalytic contribution of the sulfuric acid on the thermal decomposition process [70,71]. The presence of the sulphate groups enabled the formation of carbon residues at the end of the pyrolytic decomposition [31]. However, these residues, (*R*), could not be correlated. During heating, sulfuric acid molecules are released and facilitate the removal of the hydroxyl groups in the form of water or reacting with them through esterification, which causes the subsequent removal of sulfuric acid [77,78,79]. At this point, the thermal stability was evaluated in detail in the next section through kinetic analysis to understand the reaction mechanisms involved in the degradation process.

### 3.3. Kinetic Analysis and Apparent Activation Energy during Pyrolysis

The thermal decomposition of the CNCs was assessed from a kinetic perspective, including measurements at different heating rates of 5, 10, 20, and 30 °C·min^−1^. From these results, the zero-decomposition temperatures (*ZDT*) were gained, assessing the thermal decomposition behavior (*TDB*) when the heating rate tends to zero (*β*→0). This allows avoiding the influence of the dynamic heating program during thermogravimetric analyses [41,80,81]. For this purpose, results were fitted to the exponential relationship shown in Equation (9), where *a*, *b* and *k* are parameters of the fitting.

(9) $$TDB\left(\beta \right)=\frac{a}{1+b\cdot e^{-k\cdot \beta }}$$

**Figure 6:**
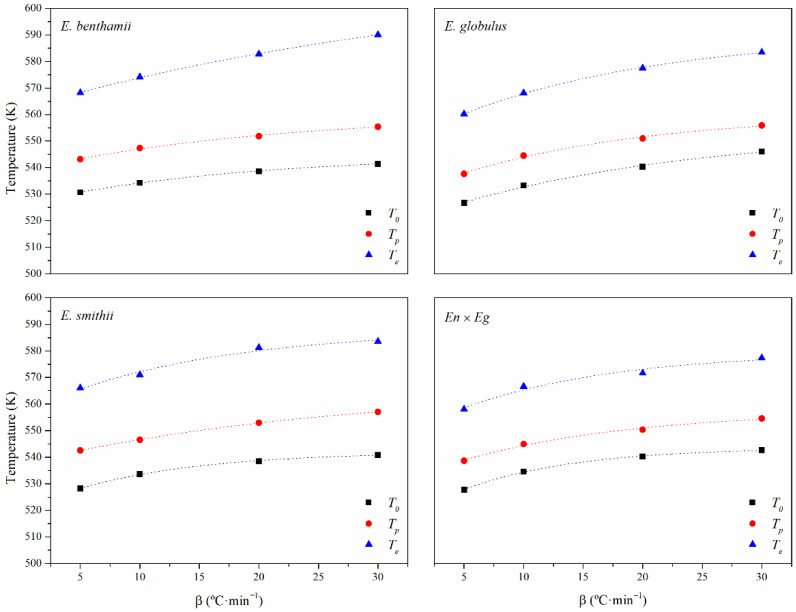
Evolution of the onset (*T_o_*), peak (*T_p_*) and endset (*T_e_*) temperatures as a function of the heating rate (*β*) of the CNCs obtained from the different eucalyptus species. Standard deviations below 2 K were omitted for the sake of clarity.

Figure 6 shows the evolution of the onset (*T_o_*), peak (*T_p_*) and endset (*T_e_*) temperatures as a function of the heating rate (*β*). Table 6 gathers the obtained values along with the fitting parameters and regression coefficients for the CNCs of all eucalyptus species. In general, the fitting lines showed a high degree of correlation (*R*^2^). Although all the *ZDT* values may give appreciated information, the technological utility of the beginning and culmination of the decomposition given by the onset and endset decomposition temperatures (*T_o_*, *T_e_*) is of great importance. When *β*→0, the value for the *T_o_* was in the range between 244 and 253 °C and decomposition finished at *T_e_* in the span from 276 to 289 °C. As observed before, the *E. benthamii* and *E. smithii* revealed higher thermal stability than the *En* × *Eg* and *E. globulus*, correlated to the presence of sulphate groups and the crystalline structure found before.

**Figure 7:**
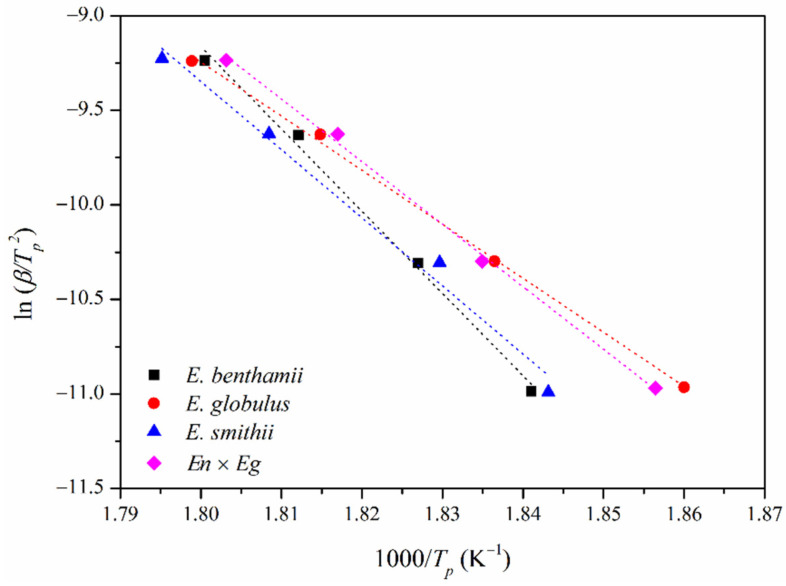
Application of the Kissinger method for the thermal decomposition of CNCs obtained from the different eucalyptus species.

Afterwards, the apparent activation energy (*Ea*) was approximated for the thermal decomposition of the CNCs, which brings valuable information to better understand its thermal behavior. For this purpose, one of the simplest approximations for calculating *Ea* was firstly carried out, as proposed by the Kissinger method [44]. Figure 7 shows the plot of ln *β*/*T_p_*^2^ vs. 1000/*T_p_*, along with the linear fitting for the different heating rates (*β*). The representation of straight lines allowed for a linear fitting, from which the apparent (*Ea*) was calculated and gathered in Table 7.

The values for the *Ea* were 362, 237, 295, and 275 kJ·mol^−1^ for the *E. benthamii*, *E. globulus*, *E. smithii*, and *En* × *Eg*, respectively, being correlated to the abovementioned thermal stability and sulphate index. The apparent activation energy was lower as the number of sulphate groups increased, corroborating that sulfuric acid catalyzed the thermal decomposition of CNCs. Moreover, these acid molecules can also promote dehydration reactions, causing the release of more molecules of water, which also contribute to catalyzing cellulose decomposition processes. Consequently, the elimination of such hydroxyl groups may reduce the presence of inter- and intramolecular hydrogen bonds, leading to a less cohesive structure and therefore to lower apparent activation energy [82].

**Figure 8:**
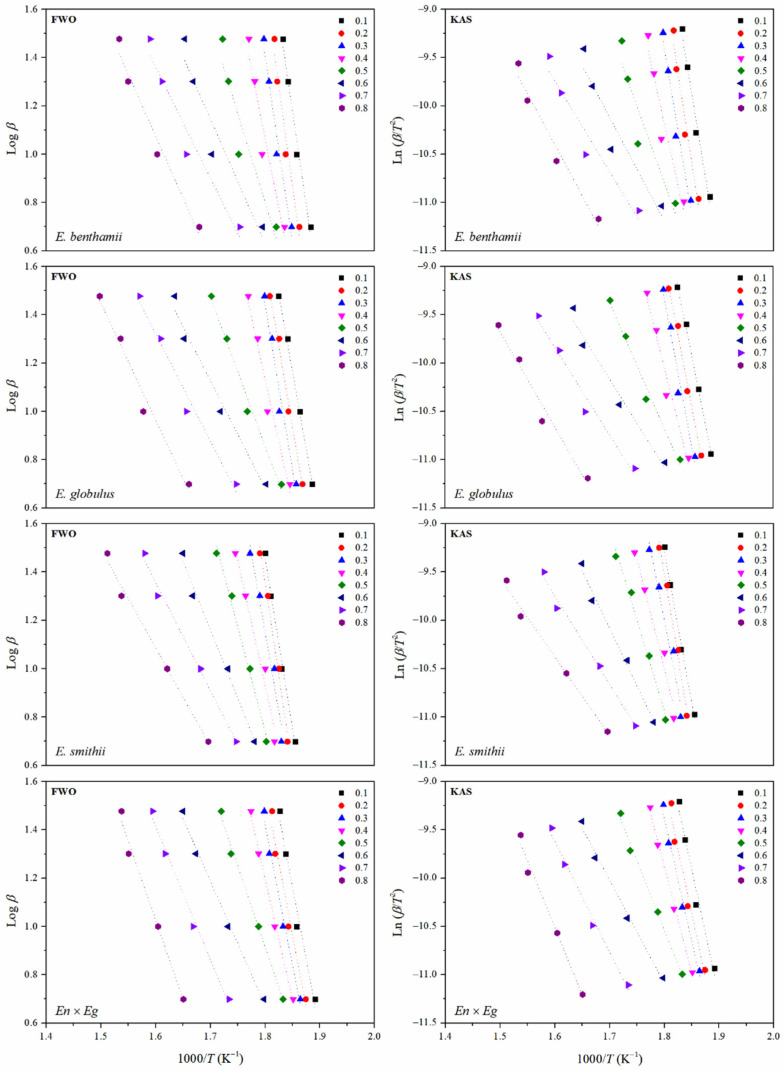
Application of Flynn–Wall–Ozawa (FWO) (**left**) and Kissinger–Akahira–Sunose (KAS) (**right**) methods for the CNCs obtained from the different eucalyptus species.

Subsequently, a deeper evaluation of the apparent *Ea* was carried out, considering the isoconversional methods proposed by Flynn–Wall–Ozawa (FWO) [45,46] and Kissinger–Akahira–Sunose (KAS) [47]. These methods allow for a more precise evaluation of the apparent activation energy as a function of conversion degree (*α*) and represent a more realistic way for understanding the variation of the *Ea* along the complete decomposition process. The obtained isoconversion plots for the CNCs obtained from the different eucalyptus species are shown in Figure 8. The FWO method involved the representation of log *β* vs. 1000/*T* while the KAS approximation plots the ln *β*/*T*^2^ vs. 1000/*T*.

**Figure 9:**
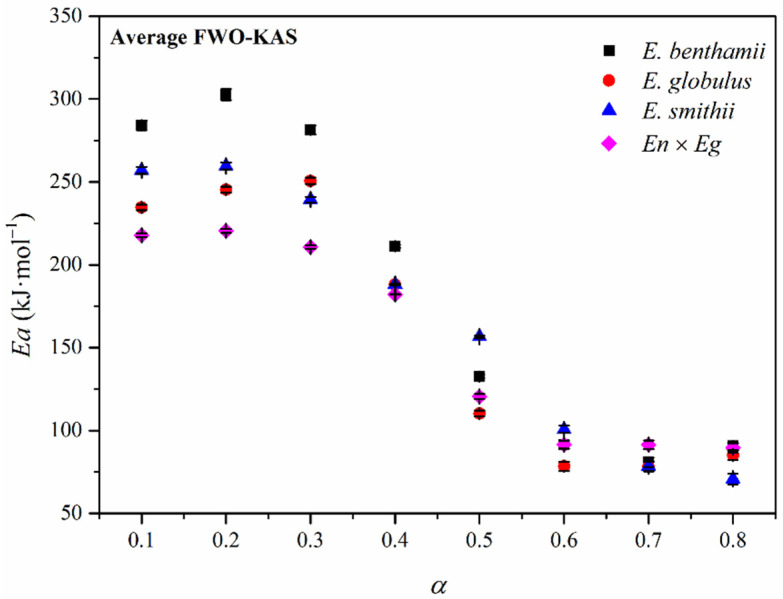
Evolution of the apparent activation energy (*Ea*) as a function of conversion calculated as an average for the FWO and KAS methods.

As gathered in Table 7, a high coefficient of determination (*R*^2^) was generally obtained during the whole conversion for both FWO and KAS methods, implying a strong correlation and validating the applied methodology for the evaluation of the apparent activation energy. Given the similar results from the FWO and KAS approximations with analogous values of activation energies with the same trend, the evolution of the *Ea* during the advance of the decomposition process was calculated and plotted in Figure 9 as an average from both methods.

As expected, the apparent activation energy smoothly varied during the thermal decomposition of CNCs, suggesting the occurrence of simultaneous degradation reactions with dissimilar natures [83]. In general, values were in the same order of magnitude as those reported in the literature for CNCs isolated from Kraft pulp, cellophane, mango seeds, or corn stalk [32,33]. However, differences can be established for CNCs with crystal structures I and II. Whereas CNCs with crystal structure II usually show an increasing trend for the activation energy as a function of the conversion degree, the CNCs with crystal structure I have been reported with a first increase in the activation energy for conversions around 0.3 followed by a decrease in conversions close to 0.6 with an increase again for higher conversions [31]. Such is the case of the CNCs assessed in this study, which all have a cellulose crystal structure I. Analogous behavior was observed with crystal structure I obtained from bleached Kraft cellulose through acid hydrolysis with sulfuric acid [31,32].

For a detailed discussion of the obtained results, the decomposition reactions at specific conversion degrees must be considered. For the low conversion range, involving the beginning of degradation up to *α* = 0.3, the volatilization of the remnant amorphous regions mainly occurs. During this stage, the sulphate groups play a crucial role, as they may have been attached prominently to amorphous domains, in which they contribute to reducing the thermal stability and activation energy. If the different eucalyptus species are compared, the *E. benthamii* showed the highest activation energy, which is again associated with the lowest sulphate index, and higher crystallinity degree and crystallite size of the CNCs [31,32]. Then, the *E. smithii*, *E. globulus*, and *En* × *Eg* showed lower values for the activation energy at such conversion degree. Afterwards, for conversions above *α* = 0.4 and up to *α* = 0.6 the apparent activation energy progressively decreased with the conversion degree for all the species, where the decomposition of the cellulose units occurs. Finally, it reaches a plateau up to *α* = 0.8, where the residue of the thermal decomposition is generated with non-relevant differences among the dissimilar CNCs.

From a technological perspective considering the preparation of polymer bionanocomposites, the apparent activation energy for the thermal decomposition of CNCs is usually higher than that of the most extended polymer matrices. This behavior may suggest that the combination of these CNCs with polymer matrices will result in higher activation energy for the overall system. However, the low percentage generally applied for reinforcing, slightly affects the overall decomposition process of the material, as demonstrated by Motta et al. with poly(styrene) (PS)/nanocellulose [84] or by Dhar et al. with poly(lactide) (PLA)/nanocellulose [85], both with filling percentages up to 1% wt, or by Coelho de Carvalho et al. with poly(hydroxybutyrate-co-3-hydroxyvalerate) (PHBV)/nanocellulose with filling percentages up to 3% wt [86]. For higher percentages, agglomeration usually occurs, and thermal stability is generally prevented due to other phenomena such as nucleation, porosity, or interphase issues, as demonstrated for some biopolymers, including PLA [87] or PHBV [86].

## 4. Conclusions

CNCs from eucalyptus Kraft pulps were prepared by sulfuric acid hydrolysis and solvent-casted films were prepared. Their evaluation exhibited some differences in terms of physico-chemical properties, especially given the dissimilar progression of the hydrolytic reaction and the incorporation of sulphate groups.

The CNCs obtained from the *E. benthamii* species were those less sulphated, with higher average diameter, less water incorporation ability with weaker interactions and higher thermal stability. Conversely, the *E. globulus* was the most sulphated of the analyzed species, giving, as a result, small hints of a slightly less crystalline morphology with a little more capability to incorporate water into their structure and also with lower thermal stability. The *E. smithii* and *En* × *Eg* revealed an intermediate behavior according to their sulphate indexes.

In the beginning of degradation up to *α* = 0.3 the volatilization of the remnant amorphous regions occurs and the sulphate groups contribute to reducing the thermal stability and activation energy. For conversions above *α* = 0.4 and up to *α* = 0.6, the apparent activation energy progressively decreased with the conversion degree for all the species. Only for conversions above *α* = 0.8 was a plateau observed and non-relevant differences among the dissimilar CNCs were found.

The evaluation of the apparent activation energy for the thermal decomposition under pyrolysis conditions served to corroborate the abovementioned performance, highlighting the reactivity or stability of CNCs from the different eucalyptus species. Particularly, it was pointed out the crucial role of the sulphate groups, that catalyzed the thermal decomposition.

## Figures and Tables

**Table 1:** Yield and composition of unbleached and bleached Kraft pulps along with CNCs obtaining yield from the different eucalyptus species.

	Unbleached Kraft Pulps				Bleached Kraft Pulps				CNC
	Yield	Glucans	Xylans	Lignin	Yield	Glucans	Xylans	Lignin	Yield
	(%)	(% wt)	(% wt)	(% wt)	(%)	(% wt)	(% wt)	(% wt)	(% wt)
*E. benthamii*	49 ± 2	73 ± 1	15 ± 1	4.3 ± 0.9	45 ± 2	73 ± 1	17 ± 1	1.3 ± 0.6	21.7 ± 1.0
*E. globulus*	57 ± 1	71 ± 1	18 ± 1	3.8 ± 0.6	53 ± 1	65 ± 1	19 ± 1	1.3 ± 0.4	14.6 ± 0.4
*E. smithii*	56 ± 2	73 ± 2	18 ± 1	3.7 ± 1.1	53 ± 2	74 ± 1	22 ± 1	<1.0	22.4 ± 1.5
*En* × *Eg*	57 ± 2	75 ± 2	19 ± 1	4.6 ± 1.3	52 ± 2	71 ± 2	22 ± 1	1.7 ± 0.7	24.9 ± 1.2

**Table 2:** Sulphate index of the CNCs obtained from the different eucalyptus species.

	Sulphate Index (%)
*E. benthamii*	0.21
*E. globulus*	0.97
*E. smithii*	0.73
*En* × *Eg*	0.85

**Table 3:** Obtaining yield along with apparent crystallinity degree (*CrI*), lateral crystallite size (*L*_002_), infrared crystallinity index (*ICI*), and infrared crystallinity ratio (*ICR*) of CNC obtained from the different eucalyptus species.

	Crystallinity Evaluation			
	*CrI* (%)	*L*_002_ (nm)	*ICI* (*A*_1430_/*A*_897_)	*ICR* (*A*_1372_/*A*_2900_)
*E. benthamii*	65.8 ± 1.3	10.9 ± 1.3	1.041	0.997
*E. globulus*	65.0 ± 1.7	10.1 ± 1.1	1.014	0.983
*E. smithii*	64.4 ± 1.6	10.1 ± 0.8	1.014	0.988
*En* × *Eg*	65.2 ± 1.9	10.0 ± 1.4	1.011	0.987

**Table 4:** Peak temperatures and enthalpies for the water release (*T_H_*_2_*_O_* and ∆*h_H_*_2*O*_) and CNC thermal decomposition (*T_cel_* and ∆*h_cel_*), as obtained from the calorimetric thermograms.

	H_2_O Release		CNC Thermal Decomposition	
	*T_free_*	∆*h_H_*_2*O*_	*T_cel_*	∆*h_cel_*
	(°C)	(J·g^−1^)	(°C)	(J·g^−1^)
*E. benthamii*	69.2 ± 0.7	57.1 ± 4.3	279.7 ± 0.2	114.0 ± 0.8
*E. globulus*	82.5 ± 2.3	84.5 ± 4.3	269.7 ± 1.8	109.2 ± 0.8
*E. smithii*	69.8 ± 0.7	49.8 ± 2.2	278.6 ± 0.2	115.2 ± 0.6
*En* × *Eg*	78.2 ± 1.9	76.1 ± 6.6	273.6 ± 0.6	120.8 ± 1.0

**Table 5:** Peak temperatures and mass loss for the release of water (*T_free_*, ∆*m_H_*_2*O*_), thermal decomposition of CNC (*T_o_*, *T_p_*, *T_e_*, ∆*m_cel_*), and final residue (*R*) obtained from the thermogravimetric analyses at different heating rates (*β*).

β		H_2_O Release		CNC Thermal Decomposition				
(°C·min^−1^)		*T_free_* (°C)	∆*m*_*H*__2__*O*_ (%)	*T_o_* (°C)	*T_p_* (°C)	*T_e_* (°C)	∆*m_cel_* (%)	*R* (%)
5	*E. benthamii*	69.5 ± 1.6	2.5 ± 0.4	260.0 ± 0.5	270.0 ± 1.4	295.1 ± 2.9	69.3 ± 0.1	28.3 ± 0.8
	*E. globulus*	69.8 ± 1.6	4.4 ± 0.7	253.6 ± 2.6	264.5 ± 1.7	287.1 ± 0.8	74.3 ± 0.2	22.3 ± 0.4
	*E. smithii*	69.1 ± 1.7	3.4 ± 0.5	255.0 ± 0.4	269.4 ± 1.3	292.9 ± 2.4	69.4 ± 2.4	26.5 ± 0.9
	*En × Eg*	70.6 ± 1.7	4.1 ± 0.3	253.8 ± 0.9	265.5 ± 2.7	284.9 ± 0.9	71.5 ± 0.9	24.1 ± 1.1
10	*E. benthamii*	61.8 ± 0.5	2.2 ± 1.0	261.2 ± 0.2	274.2 ± 0.6	301.2 ± 2.6	75.3 ± 0.1	22.5 ± 0.9
	*E. globulus*	87.9 ± 0.2	4.8 ± 1.0	260.1 ± 1.9	271.4 ± 1.4	295.0 ± 2.2	78.4 ± 1.7	16.8 ± 1.2
	*E. smithii*	61.2 ± 1.6	2.5 ± 0.4	260.5 ± 1.5	273.4 ± 1.3	297.9 ± 3.1	72.5 ± 0.7	23.1 ± 0.7
	*En × Eg*	61.1 ± 1.7	4.4 ± 0.9	261.4 ± 0.4	271.8 ± 0.2	293.5 ± 1.1	73.4 ± 1.4	24.1 ± 0.4
20	*E. benthamii*	80.9 ± 0.8	3.6 ± 0.6	265.5 ± 0.2	278.7 ± 0.9	309.6 ± 1.5	78.2 ± 0.5	18.2 ± 0.8
	*E. globulus*	83.7 ± 1.9	4.8 ± 0.9	273.2 ± 0.7	277.9 ± 0.1	304.3 ± 2.1	74.8 ± 0.2	19.4 ± 0.6
	*E. smithii*	79.0 ± 1.5	4.1 ± 1.6	265.3 ± 2.5	279.8 ± 0.2	308.1 ± 2.9	78.7 ± 2.3	14.8 ± 1.2
	*En × Eg*	89.6 ± 0.4	4.5 ± 0.1	267.1 ± 1.8	277.2 ± 2.8	298.5 ± 5.6	80.0 ± 0.4	15.9 ± 0.9
30	*E. benthamii*	83.7 ± 1.9	3.4 ± 0.2	267.3 ± 1.0	282.3 ± 1.3	316.9 ± 0.9	76.8 ± 0.1	18.8 ± 0.7
	*E. globulus*	87.9 ± 0.1	4.9 ± 0.8	272.9 ± 1.7	282.8 ± 1.5	310.3 ± 3.8	74.2 ± 0.9	20.9 ± 0.4
	*E. smithii*	81.5 ± 3.0	3.3 ± 0.9	267.7 ± 1.0	283.9 ± 0.1	310.4 ± 0.3	81.4 ± 0.7	14.8 ± 0.9
	*En × Eg*	89.7 ± 1.7	3.8 ± 0.2	269.5 ± 1.9	281.4 ± 2.5	304.2 ± 3.7	76.9 ± 0.1	19.8 ± 1.1

**Table 6:** Values of *ZDT* along with the fitting parameters and regression coefficient for the thermal decomposition of CNC (*T_o_*, *T_p_*, *T_e_*, ∆*m_cel_*) when *β*→0. Standard error was omitted for the sake of clarity.

		*ZDT*	*a*	*b*	*k*	*R* ^2^
		(°C)	(°C)			
*E. benthamii*	*T_o_*	253.0	271.5	0.04	0.06	0.998
	*T_p_*	265.5	286.9	0.04	0.05	0.991
	*T_e_*	289.0	338.7	0.09	0.03	0.998
*E. globulus*	*T_o_*	246.7	280.8	0.07	0.05	0.990
	*T_p_*	256.5	286.8	0.06	0.07	0.987
	*T_e_*	277.3	316.9	0.07	0.06	0.998
*E. smithii*	*T_o_*	246.8	268.8	0.04	0.10	0.996
	*T_p_*	270.1	292.4	0.02	0.07	0.972
	*T_e_*	283.3	314.8	0.06	0.07	0.957
*En* × *Eg*	*T_o_*	243.9	270.4	0.05	0.11	0.998
	*T_p_*	258.1	284.3	0.05	0.07	0.981
	*T_e_*	275.6	306.4	0.06	0.08	0.928

**Table 7:** Apparent activation energy (*Ea*) for the thermal decomposition of CNCs obtained from the different eucalyptus species.

		Kissinger		FWO		KAS	
		*Ea*	*R* ^2^	*Ea*	*R* ^2^	*Ea*	*R* ^2^
		(kJ·mol^−1^)		(kJ·mol^−1^)		(kJ·mol^−1^)	
*E. benthamii*	0.1	361.5	1.00	281.3	0.98	287.1	0.98
	0.2			299.4	0.97	306.0	0.96
	0.3			278.6	0.97	284.0	0.97
	0.4			210.2	0.93	212.0	0.93
	0.5			133.9	0.89	131.5	0.88
	0.6			93.8	0.92	89.1	0.91
	0.7			83.6	0.95	78.0	0.94
	0.8			93.3	0.97	87.8	0.97
*E. globulus*	0.1	237.6	0.95	233.1	1.00	236.3	1.00
	0.2			243.6	0.99	247.3	0.99
	0.3			248.7	0.97	252.7	0.97
	0.4			187.8	0.97	188.4	0.97
	0.5			112.0	0.99	108.5	0.99
	0.6			81.2	0.98	75.7	0.97
	0.7			81.1	0.98	75.3	0.97
	0.8			88.1	0.98	236.3	0.97
*E. smithii*	0.1	295.2	1.00	254.9	0.99	259.2	0.99
	0.2			257.6	1.00	261.9	1.00
	0.3			237.8	0.97	241.0	0.97
	0.4			188.0	0.98	188.5	0.97
	0.5			157.2	0.99	156.0	0.99
	0.6			102.9	0.99	98.5	0.99
	0.7			81.4	0.99	75.6	0.99
	0.8			74,0	0.99	67.5	0.99
*En* × *Eg*	0.1	274.9	1.00	216.6	0.98	219.0	0.98
	0.2			219.3	0.97	221.8	0.97
	0.3			209.8	0.99	211.7	0.99
	0.4			182.0	1.00	182.4	1.00
	0.5			122.0	1.00	119.0	0.99
	0.6			93.9	0.99	89.1	0.99
	0.7			94.0	1.00	88.9	1.00
	0.8			92.4	0.99	86.7	0.99

## Data Availability

Data is contained within the article.
